# The “Trunk sign”: A novel X-ray sign in galactography of patients with nipple discharge suggesting malignancy

**DOI:** 10.1097/MD.0000000000034589

**Published:** 2023-08-11

**Authors:** Tujin Wu, Kai Zhang, Yawen Wang, Rong Ma

**Affiliations:** a Department of Thyroid and Breast Surgery, Xiamen Humanity Hospital Fujian Medical University, Xiamen, China; b Department of Breast Surgery, General Surgery, Qilu Hospital of Shandong University, Jinan, China.

**Keywords:** breast cancer, diagnosis, galactography, nipple discharge

## Abstract

The etiology of nipple discharge is often unclear, and there are few studies exploring diagnostic approaches of nipple discharge. Galactography is a common method for clinical diagnosis of patients with nipple discharge. Therefore, this study aimed to evaluate the use of galactography in differentiating between benign and malignant lesions in patients with nipple discharge. A retrospective study of 161 patients with nipple discharges, who were evaluated with galactography and underwent surgery in Qilu Hospital of Shangdong University between January 2018 and December 2019, was conducted. Baseline characteristics were obtained from their electronic records including age, menstruation status, physical examination, galactography, cytology, and pathology. There were 110 cases of benign disease, 12 cases of high-risk disease, and 39 cases of malignant disease. With respect to benign diseases there were 26 (23.6%) patients with hyperplasia and ductal ectasia, and 94 (76.4%) with intraductal papilloma. With respect to high risk diseases, there were 2 (16.7%) patients with atypical intraductal papilloma and 10 (83.4%) with atypical hyperplasia. With respect to malignant lesions, 19 (48.7%) patients had intraductal carcinoma, 4 (10.3%) had solid papillary carcinoma, and 16 (41.0%) had invasive carcinoma. The significant findings of our study are as follows: patients with malignant diseases had a higher proportion of concomitant masses (74.4% vs 41.7% vs 22.7%, *P* < .001), positive spill cytology (51.3% vs 41.7% vs 2.7%, *P* < .001), and trunk signs (71.8% vs 33.3% vs 10.9%, *P* < .001). A forest plot revealed that trunk signs were related to an increased risk of malignant diseases in almost all the subgroups. Galactography is important for the differential diagnosis of benign and malignant lesions in nipple discharge, and the “Trunk sign” is an important radiographic sign of malignant lesions. Combining galactography with other methods is advisable to improve the accuracy of diagnosis in patients with nipple discharge.

## 1. Introduction

Nipple discharge, the second most common clinical symptom among surgical patients, is a common complaint among women with breast disease.^[1]^ Nipple discharge can be divided into physiological and pathological types. Physiological nipple discharge is usually bilateral, colorless, and involves multiple ducts. Pathological nipple discharge is generally unilateral, uniductal, bloody, spontaneous, and 85% to 95% is caused by intraductal papilloma, ductal ectasia, and papillomatosis.^[2]^ However 5% to 15% of pathological nipple discharge is caused by malignant breast disease, which is difficult to identify as benign nipple discharge in clinical practice.

The etiology of nipple discharge is often unclear, and there is no accepted examination method that can differentiate between benign and malignant nipple discharge.^[3,4]^ With gradual improvements in medical technology, the cause of nipple discharge should be evaluated using medical history, physical examination, mammography, ultrasonography, and cytological tests.^[5,6]^ However, negative results can be found because of the absence of a breast mass or a small breast mass in such patients. Therefore, there are no consensus methods for the diagnosis of patients with nipple discharge.

Galactography is a simple, safe, and effective method for locating, diagnosing, and directing the surgical excision of intraductal lesions. It was first used clinically in patients with unilateral nipple discharge on mammography, after injecting a contrast agent. In 1960, with the discovery of water-soluble contrast media, galactography was widely used in the diagnosis of patients with nipple discharge.^[7]^ This is particularly important when negative results are obtained by mammography and ultrasonography. Although galactography is a fundamental technique for detecting and locating intraductal abnormalities, its use is still controversial; thus, the purpose of this study was to evaluate the use of galactography in identifying benign and malignant breast diseases.

## 2. Methods

This retrospective study used data from patients with nipple discharge, who were evaluated with galactography and underwent surgery in the Qilu Hospital of Shangdong University between January 2018 and December 2019. The exclusion criteria were as follows: male patients, failure of galactography, and incomplete data based on previous study.^[8]^ A total of 161 female patients were enrolled in this study. Baseline characteristics were obtained from the electronic records, including age, menstruation status, physical examination, galactography, cytology, and pathology. Two independent reviewers (T.J.W. and R.M.) searched PubMed and Web of Science from database inception to September 01, 2022 to select information from the literature. This study was reviewed and approved by the Ethics Committee of the Qilu Hospital of Shangdong University. We obtained informed consent from all the participants included in this research. All study procedures were performed in accordance with the Helsinki Declaration of 1964 and its later versions.

Galactography was performed after cytological examination of nipple discharge with reference to previous study.^[9]^ We prepared a lacrimal duct irrigation needle using a 1 ml empty needle, gauze, alcohol, lamp, and cotton swab before galactography. Firstly, 0.2 mL iodiproamine injection 300 was extracted with a 1 mL empty needle. We then appropriately squeezed out a certain amount of the contrast agent, drained the lacrimal duct to rinse the air in the needle, or used a 1 mL empty needle head. Patients were seated, and an alcohol cotton swab was used to gently sterilize the nipples before galactography and to stimulate the dilation of the leaking milk duct to facilitate needle insertion. Then the needle in the researcher’s right (dominant) hand was gently inserted through the nipple hole, and when there was a sensation of a slight breakthrough, we asked if the patient felt pain. If the patient did not experience pain during the needle insertion process, it was assumed to indicate that the needle had entered the milk duct. We then injected the contrast agent slowly and gently, stopping when the patient felt uncomfortable or had contrast agent overflow from the milk pore.^[10–12]^ The needle was then gently removed, and the contrast agent was rubbed off the surface of the patient’s skin and nipple with alcohol. Bilateral or contrast-side mammograms were immediately performed using a German VENUS X-ray machine.

The pathological results were classified as benign (ductal ectasia, intraductal papilloma, and papillomatosis), high-risk disease (atypical hyperplasia and intraductal papilloma with atypical hyperplasia), or malignant disease (invasive breast cancer, intraductal carcinoma, solid papillary carcinoma).

Analyses were performed using SPSS (Statistical Product and Service Solutions) statistical software and GraphPad Prism 5. Statistical significance was set at *P* < .05. Pearson’s chi-squared test was used for categorical variables. Univariate and multivariate analyses were performed to determine malignancy-related elements.

## 3. Results

### 3.1. Baseline characteristics

**Figure 1.**
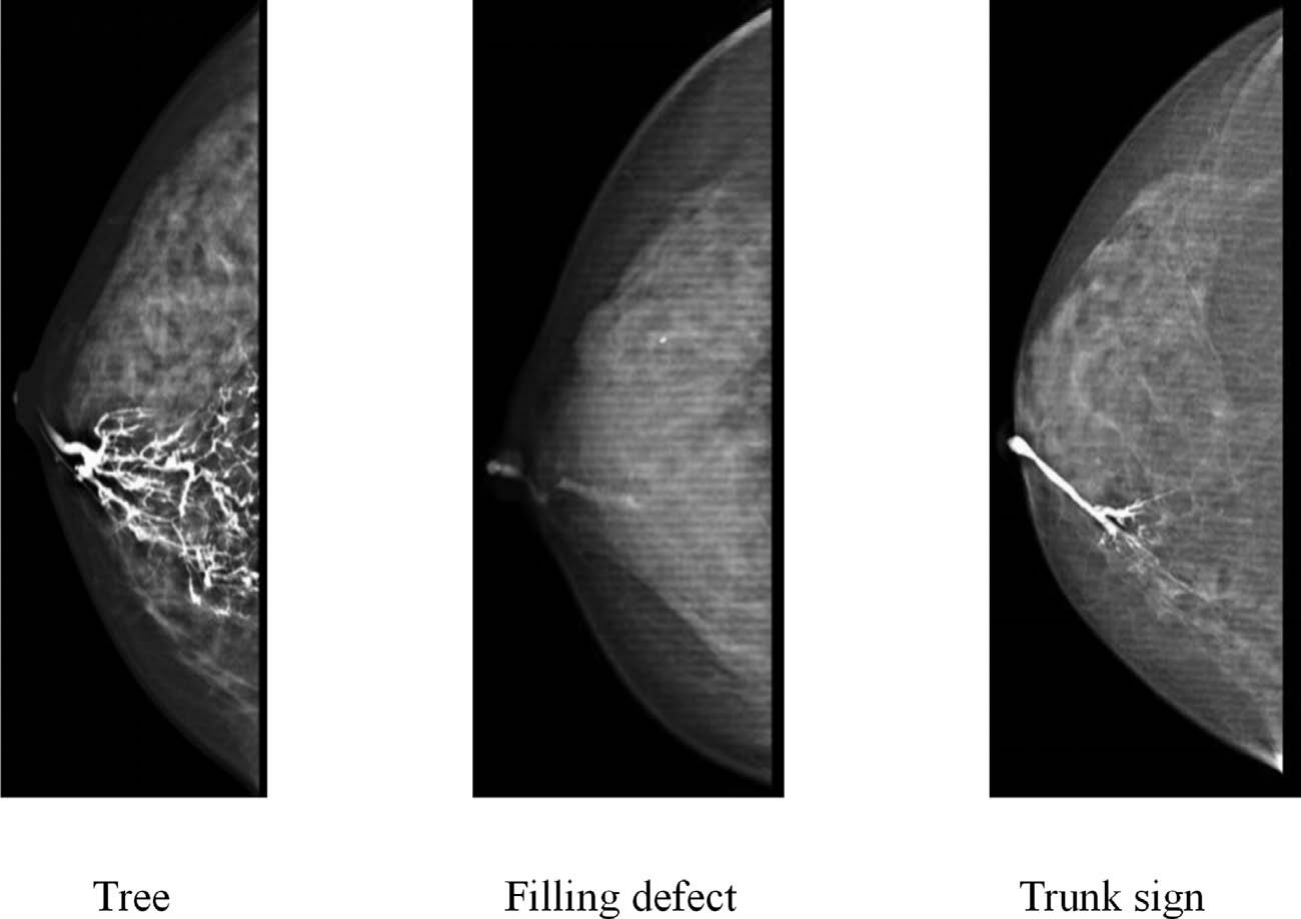
Classification of galactography.

During the study period, 172 patients with nipple discharge were identified. We excluded 11 patients due to the following reasons: being male (n = 1), failure of galactography (n = 9), and incomplete data (n = 1). We obtained data from 161 patients. Of these 110 patients had benign diseases, 12 patients had high-risk diseases, and 39 patients had malignant diseases. In the benign disease group there were 26 (23.6%) patients with hyperplasia and ductal ectasia, 94 (76.4%) with intraductal papillomas, In the high-risk group there were 2 (16.7%) patients with atypical intraductal papilloma and 10 (83.4%) with atypical hyperplasia. In the malignancy group there were 19 (48.7%) patients with intraductal carcinoma, 4 (10.3%) with solid papillary carcinoma, and 16 (41.0%) with invasive carcinoma. The results of galactography were classified as “trunk sign,” filling defect, or tree (Fig. 1). Patients with malignant diseases had a higher proportion of concomitant masses (74.4% vs 41.7% vs 22.7%, *P* < .001), positive spill cytology (51.3% vs 41.7% vs 2.7%, *P* < .001), and trunk signs (71.8% vs 33.3% vs 10.9%, *P* < .001). There were no significant differences in age, menstruation status, location, disease time, nipple discharge color, or papillary pores (Table 1).

**Table 1:** Baseline characteristics.

	Benign (n = 110)	High-risk (n = 12)	Malignant (n = 39)	*P*
Age				
≤50	88 (80.0)	11 (91.7)	27 (69.2)	.189
>50	22 (20.0)	1 (8.3)	12 (30.8)	
Pausimenia				
Yes	24 (21.8)	2 (16.7)	12 (30.8)	.444
No	86 (78.2)	10 (83.3)	27 (69.2)	
Location				
Left	58 (53.7)	5 (41.7)	16 (40.0)	.394
Right	52 (46.3)	7 (58.3)	23 (60.0)	
Disease time				
1–6 mo	59 (53.6)	7 (58.4)	16 (41.0)	.225
6–12 mo	13 (11.8)	2 (16.7)	11 (28.2)	
12–24 mo	8 (7.3)	1 (8.3)	5 (12.8)	
>24 mo	30 (27.3)	2 (16.7)	7 (17.9)	
Nipple discharge color				
Clear	5 (4.5)	1 (8.3)	3 (7.7)	.335
White	2 (1.8)	0 (0)	1 (2.6)	
Yellow	53 (48.2)	4 (33.3)	10 (25.6)	
Bloody	50 (45.5)	7 (58.3)	25 (64.1)	
Papillary pores				
Single	91 (82.7)	8 (66.7)	36 (92.3)	.092
Porous	19 (17.3)	4 (33.3)	3 (7.7)	
Concomitant mass				
No	85 (77.3)	7 (58.3)	10 (25.6)	<.001
Yes	25 (22.7)	5 (41.7)	29 (74.4)	
Spill cytology				
Negative	107 (97.3)	7 (58.3)	19 (48.7)	<.001
Positive	3 (2.7)	5 (41.7)	20 (51.3)	
GL-X				
Trunk	12 (10.9)	4 (33.3)	28 (71.8)	<.001
Filling defect	35 (31.8)	2 (16.7)	4 (10.3)	
Tree	63 (57.3)	6 (50.0)	7 (17.9)	

### 3.2. Univariate and multivariate analyses

**Figure 2.**
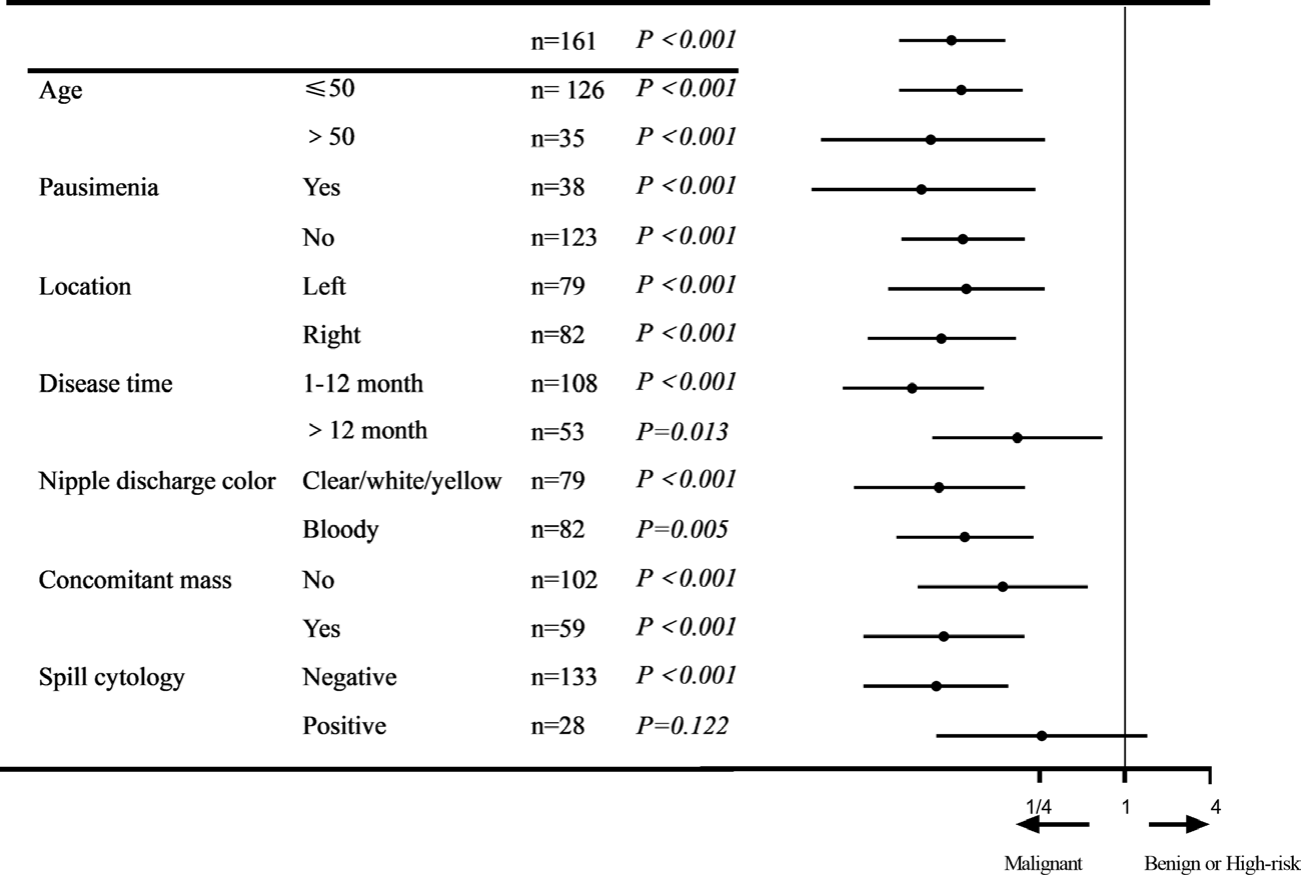
Odds ratio for malignant breast diseases. Forest plot evaluating the predictive effect of galactography on malignant breast diseases.

Among the 161 patients enrolled in this study, there were 76 cases with 3 signs: 63 cases (82.9%) of benign lesions, 6 cases (7.9%) of high-risk lesions, and 7 cases (9.2%) of malignant lesions; 41 cases with filling defect signs: 35 benign lesions (85.4%), 2 high-risk lesions (4.9%), and 4 malignant lesions (9.8%); 44 cases with trunk signs: 12 benign lesions (27.3%), 4 borderline lesions (9.1%), and 28 malignant lesions (63.6%). Univariate and multivariate analyses identified a disease time of 1 to 6 months, concomitant mass, positive spill cytology, and trunk sign as independent risk factors for malignant diseases (Table 2). A forest plot revealed that the trunk sign was related to an increased risk of malignant diseases in almost all the subgroups (Fig. 2).

**Table 2:** Univariate and multivariate analyses.

Variable	OR	95% CI	*P* value
Univariate analysis			
Age (≤50/>50)	1.913	0.845–4.332	.120
Pausimenia (Yes/no)	0.609	0.272–1.365	.229
Location (Left/right)	1.535	0.740–3.186	.250
Disease time			
1–6 mo	Ref	Ref	Ref
6–12 mo	3.025	1.169–7.826	.022
12–24 mo	2.292	0.675–7.778	.184
>24 mo	0.902	0.338–2.412	.838
Nipple discharge color			
Clear	Ref	Ref	Ref
White	1.000	0.063–15.988	1.000
Yellow	0.351	0.075–1.637	.183
Bloody	0.877	0.203–3.790	.861
Papillary pores (Single/porous)	0.359	0.102–1.267	.111
Concomitant mass (No/yes)	8.893	3.884–20.365	<.001
Spill cytology (Negative/positive)	15.000	5.784–38.901	<.001
GL-X			
Trunk	Ref	Ref	Ref
Filling defect	0.062	0.019–0.205	<.001
Tree	0.058	0.022–1.156	<.001
Multivariate analysis			
Disease time			
1–6 mo	Ref	Ref	Ref
6–12 mo	5.496	1.174–25.733	.031
12–24 mo	3.431	0.493–23.883	.213
>24 mo	2.852	0.679–11.972	.152
Concomitant mass (No/yes)	9.725	2.842–33.276	<.001
Spill cytology (Negative/positive)	14.894	4.024–55.123	<.001
GL-X			
Trunk	Ref	Ref	Ref
Filling defect	0.090	0.021–0.381	.001
Tree	0.098	0.028–0.339	<.001

## 4. Discussion

Nipple discharge, breast pain, and breast masses are the 3 main symptoms of breast cancer. The incidence of nipple discharge is approximately 3% to 6%.^[13]^ Although most nipple discharges are caused by benign lesions, about 5% to 10% are caused by breast cancer.^[14]^ The etiology of pathological nipple discharge is often unclear and there is no universally recognized uniform standard for diagnosis. Galactography is considered to be the most effective clinical test for nipple discharge. Breast masses of more than 1 cm can be found by physical examination, but affected areas that have not formed detectable masses can be found by galactography, especially in patients with pathological nipple discharges. When mammography and color Doppler results are negative, galactography is particularly important.^[15–18]^ To the best of our knowledge, the present study is the first to demonstrate the morphological use of galactography to detect breast cancer. Our study revealed that the trunk sign was associated with an increased risk of breast cancer, which enriched the function of galactography.

Although various methods are available for the examination of nipple discharge, there is no unified method for the identification of benign and malignant diseases. Many experts believe that galactography can indicate a malignancy. Signs such as catheter dilation, multiple filling defects, irregular guide walls, and catheter blockage indicate malignancy. Juan et al^[19]^ divided duct galactography into 5 categories by studying X-ray signs of duct dilation, filling interruption, tube wall distortion, and other details. Taking the results of pathology after surgery as the gold standard, they found that the galactography classification system has a good correlation with postoperative pathology, which can be used for the differential diagnosis of benign and malignant tumors. This classification method can be widely used in clinics.^[8,20]^ The difference between our study and the above one is that we categorized the overall morphology of galactography, and evaluated whether this classification system is meaningful for the differential diagnosis of benign and malignant diseases. Our results showed that “filling defect sign” and “tree sign” were mainly found in benign lesions, and with the decrease in the proportion of high-risk lesions, the proportion of malignant lesions was lowest. In contrast, “trunk sign” was the main galactography manifestation in breast cancer, followed by high-risk lesions and least-benign lesions. Multivariate analysis revealed that trunk signs were associated with an increased risk of breast cancer. To the best of our knowledge, we were the first to propose this classification method. It is simple and easy to be identified. The “trunk sign” has a promising future in distinguishing breast cancers with further study in comparison with existing therapeutics. What’s more, the field of biomedicine is advancing rapidly, and the nanoscopic materials are being investigated in cancer diagnosis.^[21,22]^ Higher sensitivity may be achieved with these new materials used in galactography technology.

Some experts have proposed that papillary discharge cytology is a good method for identifying patients with benign and malignant nipple discharge. Cytological examination of nipple discharge is an effective method, and we hope to identify benign and malignant lesions by identifying cancer cells in the nipple discharge. Breast cancer often coexists with intraductal papilloma; intraductal papilloma is often located near the nipple, which influences cytological sensitivity.^[23]^ The cytological sensitivity of nipple discharge is low, and some researchers believe that only positive results are clinically significant.^[24]^ Ciatto et al^[25]^ conducted a cytological examination of 3687 patients with nipple discharge and found that the detection rate was 45%, suggesting that patients with non-bleeding nipple discharge were unsuitable for cytological examination. Clinically, repeated tests are required to improve the sensitivity and specificity. In the present study, a forest plot revealed that the trunk sign was related to an increased risk of malignant diseases in almost all subgroups, especially in patients with negative spill cytology. It is thus advisable to combine cytological examination with galactography to improve the diagnosis of nipple discharge.

Pathological nipple discharge is usually bloody, but may also be yellow, green, purulent, or white. A bloody nipple discharge is usually associated with breast cancer. Whether the color of nipple discharge can be used to distinguish benign from malignant is controversial. Some experts have found that nipple discharge color is related to the nature of the disease,^[26]^ believing that patients with bloody or dark nipple discharge have a higher risk of breast cancer.^[27]^ However, some experts have proposed that the color of nipple discharge is not meaningful for the differential diagnosis of breast cancer, and bloody nipple discharge does not increase the risk of breast cancer.^[28]^ In the present study, no significant difference was found in the color of nipple discharge between the different types of breast diseases. Therefore, nipple discharge color cannot be used in the differential diagnosis of benign and malignant diseases, and other nipple discharge colors cannot completely rule out the possibility of breast cancer.

This study had some limitations. This sample size was relatively small. Moreover, this study was limited by its retrospective design. Second, the nipple discharge color was distinguished by the naked eye, which was divided into blood color, white, yellow, and clear. Finally, this study was a single-center sample study that needs to be combined with multiple hospitals for further comparative studies.

## 5. Conclusion

We demonstrated that galactography is important for the differential diagnosis of benign and malignant breast diseases, and that “trunk sign” is a crucial X-ray sign of malignant lesions. Combining galactography with other methods is advisable to improve the diagnosis of patients with nipple discharge. Further investigations should be conducted in order to develop personalized diagnostic schemes.

## Acknowledgments

The authors thank all colleagues and nurses who provided care to the patients in this study.

## Author contributions

**Conceptualization:** Tujin Wu, Kai Zhang, Yawen Wang, Rong Ma.

**Data curation:** Tujin Wu, Kai Zhang, Yawen Wang, Rong Ma.

**Formal analysis:** Tujin Wu, Yawen Wang, Rong Ma.

**Funding acquisition:** Kai Zhang, Yawen Wang, Rong Ma.

**Investigation:** Tujin Wu, Kai Zhang, Rong Ma.

**Software:** Tujin Wu, Kai Zhang, Yawen Wang.

**Writing – original draft:** Tujin Wu, Rong Ma.

**Writing – review & editing:** Tujin Wu, Rong Ma.
